# Catastrophic costs among drug-susceptible tuberculosis households in the Guangxi Zhuang Autonomous Region, China: determinants and economic impact evaluation

**DOI:** 10.3389/fpubh.2026.1872600

**Published:** 2026-07-24

**Authors:** Tengyan Wu, Li Yang, Suosu Wei, Zong Tang, Pinghua Zhu, Yanlong Wu, Xiao Chen, Guiyin Lao, Yi Man, Shuanglin Li, Qiongyun Pei

**Affiliations:** 1 School of Information and Management, Guangxi Medical University, Nanning, China; 2 School of Public Health, Peking University, Beijing, China; 3 Guangxi Academy of Medical Sciences, People’s Hospital of Guangxi Zhuang Autonomous Region, Nanning, China; 4 TB Prevention and Control Institute of Yulin City, Yulin, Guangxi, China; 5 People’s Hospital of Tiandeng County, Tiandeng, Guangxi, China

**Keywords:** catastrophic costs, cross-sectional survey, influencing factors, out-of-pocket payments, tuberculosis (TB)

## Abstract

**Background:**

Tuberculosis (TB) remains a major poverty-associated infectious disease worldwide. This study is the first to quantify the incidence and determinants of catastrophic costs among drug-susceptible TB patients in Guangxi, providing critical evidence for developing poverty alleviation policies and promoting equitable TB care access.

**Methods:**

The study included 250 drug-susceptible TB patients who were registered and completed treatment between June 2023 and June 2024 in local designated TB hospitals across three counties in Guangxi with different per capita disposable income. Using structured questionnaires, we collected comprehensive data on socioeconomic characteristics and TB-related direct/indirect costs. All statistical analyses were performed using SPSS 27.0.

**Results:**

During the pre-diagnosis stage, post-diagnosis stage, and the entire course of TB care, the median out-of-pocket payments per TB patient were 2501.5 RMB, 2323.8 RMB, and 6323.7 RMB, respectively. Direct medical costs constituted the largest proportion of total costs at each stage of TB care. The incidence of catastrophic costs due to TB was 45.6% (114/250). The results of the $x^{2}$ test showed that there were statistically significant differences in the incidence of catastrophic costs according to education levels, annual household income, medical insurance type, and number of hospitalization days after diagnosis (*p* < 0.05). The results of the binary logistic regression analysis showed that patients with annual household income ≦20,000 RMB and those with more than 7 hospitalization days after diagnosis had a higher risk of catastrophic costs due to TB, and the ORs (95%CI) were 34.168 (12.742–91.625) and 7.488 (2.474–22.664), respectively.

**Conclusion:**

Most costs were incurred before diagnosis and were primarily direct medical costs. To reduce catastrophic costs, we recommend targeted TB health education, enhanced screening/treatment monitoring, improved multi-tiered insurance coverage, support for rural low-income populations, early cost-warning systems, and sustainable TB-related poverty prevention mechanisms.

## Background

Tuberculosis (TB) remains a critical global public health challenge with profound socioeconomic implications, particularly affecting impoverished populations. TB-affected households facing catastrophic costs have emerged as a significant barrier to sustainable development in high-burden regions. Eliminating catastrophic TB costs constitutes a fundamental pillar of the WHO End TB Strategy (1, 2). Current evidence demonstrates substantial heterogeneity in TB-related economic burden across geographical regions and demographic groups, with vulnerable populations disproportionately affected (3–5).

As a high-burden country accounting for approximately 6.8% of global tuberculosis cases (ranking third worldwide) (1), China faces significant challenges in mitigating the economic impact of TB. Reducing catastrophic costs among TB-affected households represents a critical component of national efforts to eliminate TB-associated morbidity, alleviate poverty, and achieve the targets of both the WHO End TB Strategy and China’s Healthy China 2030 objectives (6). The Chinese government has implemented comprehensive TB control measures, including free treatment policies and enhanced medical insurance coverage (7). Specifically, the state provides free sputum smear and chest X-ray examinations for presumptive TB cases and offers free first-line anti-TB drugs and basic examinations during treatment for active pulmonary TB patients. In terms of medical insurance, TB has been included in outpatient special disease or chronic disease management schemes in many regions, with inpatient reimbursement rates within the policy scope remaining stable at approximately 70% in some areas (6). In addition, China has continuously optimized its TB service delivery system, transitioning from a Centers for Disease Control and Prevention (CDC)-led model to a “trinity” model, in which the CDC is responsible for planning and management, designated medical facilities undertake diagnosis and treatment, and primary healthcare facilities handle patient follow-up (7). Through government leadership and multi-sectoral collaboration, this model has achieved a full-chain, closed-loop management system from case detection to cure and has basically enabled ordinary TB diagnosis and treatment to be completed within counties. However, this model still has shortcomings in alleviating patients’ financial burden. On the one hand, due to different operational mechanisms between designated hospitals and the CDC, some hospitals exhibit non-standardized practices and over-treatment, which drive up patient costs. On the other hand, under the current medical insurance system, the outpatient reimbursement rate and cap for TB patients remain limited. Moreover, some costs incurred before diagnosis, direct non-medical costs such as transportation and accommodation, and indirect costs such as income loss due to work absence are not covered by insurance (4). As a result, the disease-related economic burden on patients remains substantial, and previous studies have shown that this burden is still serious in some regions (2, 4).

The Guangxi Zhuang Autonomous Region (Guangxi), an economically underdeveloped region in southwestern China, bears a disproportionately high TB burden, with surveillance data from the past decade consistently showing that TB ranks among the top two reported Class A/B infectious diseases in the region and that Guangxi remains among China’s seven highest-burden provinces (8, 9). Since 2003, the region has implemented WHO-recommended TB control strategies through the World Bank/UK Grant-funded project, providing free diagnostic services (including chest radiography and sputum smear microscopy) and first-line anti-tuberculosis medications. Other medical expenses have been reimbursed through the national basic medical insurance system, and the TB control service system is being optimized continuously (10). However, this package of policy measures is still not enough to solve the economic burden on TB patients (11). There is an urgent need to comprehensively evaluate the economic burden on TB patients, but research on catastrophic costs due to TB is scarce. The purpose of this study was to investigate the treatment costs of TB patients and to analyze the current situation of catastrophic costs due to TB and their associated factors. The findings will inform targeted poverty alleviation strategies in Guangxi while providing transferable evidence for other high-burden regions in China.

## Materials and methods

### Study areas

A purposive sampling method was adopted to select Y County in Yulin City, which had a higher per capita disposable income of 40,755 RMB, and H County in Hechi City and L County in Chongzuo City, which had lower per capita disposable incomes of 27,331 RMB and 20,371 RMB, respectively, according to the 2023 per capita disposable income data reported in the Guangxi Statistical Yearbook. All three counties had designated TB hospitals. Specifically, in Y County in Yulin City, the designated TB hospitals were the local TB Control Institute and the Red Cross Hospital. In H County in Hechi City and L County in Chongzuo City, the designated hospitals were the respective county People’s Hospitals. All patients included in this study were TB patients registered and treated at these designated TB hospitals in their respective counties.

### Study design

A cross-sectional study was conducted among 250 TB patients with TB who were registered at the designated TB hospitals in the three counties of Guangxi. A two-stage sampling approach was used. First, cluster sampling was applied to select survey sites (counties and designated TB hospitals). Second, convenience sampling was used to recruit eligible TB patients who were registered and had completed anti‑TB therapy between June 2023 and June 2024 for a retrospective questionnaire survey. Counties were treated as clusters, and the sample sizes for each county were proportionally allocated based on the total number of patients who were registered and completed anti-TB therapy during the study period. Accordingly, 100 participants were recruited from Y County (Yulin City), 50 cases from H County (Hechi City), and 100 from L County (Chongzuo City). All participants signed an informed consent form.

The inclusion criteria were as follows: ① Treatment classification: patients who received either initial treatment or retreatment, ② Registration classification: patients who were registered as cured or having completed treatment, ③ age ≥18 years, ④ provision of written informed consent, and ⑤ patients with clear awareness. The exclusion criteria were as follows: ① Drug-resistant TB patients; ② extrapulmonary TB patients; ③ patients with severe liver or kidney dysfunction, serious heart or lung diseases, mental disorders, AIDS, or cancer; ④ patients with visual, hearing, and speech impairments; and ⑤ patients with incomplete clinical or economic information.

### Data collection

A questionnaire was used to collect the medical information of TB patients in this study. The questionnaire was developed with reference to the survey instrument formulated by the Chinese Center for Disease Control and Prevention (CDC) for the comprehensive evaluation of the National Tuberculosis Prevention and Control Plan (2016–2020) (4). The final version was developed after discussion and modification by experts in TB prevention and control in Guangxi. The questionnaire was initially pre-tested among 10 TB patients and subsequently revised based on their feedback. This group of TB patients was later excluded from the study.

The questionnaire survey was conducted by trained investigators through face-to-face interviews or telephone follow-up. The questionnaire was divided into four sections. The first section collected information on participants’ sociodemographic characteristics, including gender, age, ethnicity, occupation, etiological examination results, and treatment classification. The second section focused on pre-diagnosis costs, including the courses and the costs of medical treatment from the onset of suspicious symptoms to the identification of TB, excluding the costs of free examination items. The third section focused on post-diagnosis costs, including the courses and the costs of medical treatment and follow-up visits at designated TB hospitals, excluding the costs of free tests and free medicines. Lastly, the fourth section focused on the economic conditions and impacts, including the income of TB patients and their families during the course of diagnosis and treatment, as well as other impacts on daily life. The questionnaire included multiple-choice questions, single-choice questions, and fill-in-the-blank questions.

To ensure data quality, the information collected through the questionnaire was verified by data auditors according to the TB Information System of the Chinese CDC and the Hospital Information System at the end of the investigation, and the investigator was contacted to confirm and supplement the missing items.

### Ethical approval and confidentiality

Ethical approval for this study was obtained from the Research Ethics Board of Health of Guangxi Medical University, China (No: 2023KY0033). All participants signed an informed consent form before participating in this study. Survey data were securely stored in password-protected, encrypted folders on the computers of the principal investigators and co-investigators.

### Definitions

#### Total costs

It refers to all expenses incurred throughout the course of TB care, from the onset of suspicious symptoms to the completion of treatment. These costs exclude free tests and free medicines provided through the national program but include payments made through national medical insurance and other subsidy programs. Total costs consist of direct costs and indirect costs, where direct costs are the sum of direct medical costs and direct non-medical costs.

#### Total out-of-pocket payments

It refers to the sum of direct medical, direct non-medical, and indirect costs that remain after deducting reimbursements from national medical insurance and other subsidies from the total costs.

#### Direct medical costs

It refers to the medical costs associated with TB care from the onset of suspicious symptoms to the completion of treatment, including costs related to outpatient and hospitalization registration, examinations, and treatment.

#### Direct non-medical costs

It refers to the ancillary costs incurred by TB patients and their accompanying family members from the onset of suspicious symptoms to the completion of treatment, including transportation, accommodation, food, and other related expenses.

#### Indirect costs

It refers to the social wealth loss experienced by TB patients and their family members due to work absence caused by illness throughout the entire course of diagnosis and treatment. It is estimated using the human capital method (12), as follows: indirect economic burden = days of work missed by patients × daily income of patients + days of work missed by accompanying family members × daily income of family members.

#### Delay in diagnosis

It refers to the time interval between the first healthcare-seeking visit and the identification of TB, which is ≥14 days.

#### Annual household incomes

It refers to the income earned from labor or services, the sale of goods and assets, and financial investments by patients and their family members during the year. In this study, annual household income data were collected through patient self-reporting.

#### Catastrophic costs

It refers to total out-of-pocket payments for TB care that exceed 20% of a patient’s annual household income (12).

#### Incidence of catastrophic costs

It refers to the number of patients experiencing catastrophic costs/total number of patients investigated ×100%.

### Data analysis

The software EpiData3.1 was used to establish the database, and the software SPSS 27.0 (IBM Corporation, Armonk, State of New York) was used to carry out statistical analysis. Frequencies and percentages (%) were used for the statistical description of categorical data, while medians and quartiles, expressed as M (Q1, Q3), were used for measurement data that did not conform to a normal distribution, such as cost data. The $x^{2}$ test was used for univariate analysis. Binary logistic regression analysis was performed for the multivariable analysis. A *p*-value of < 0.05 indicated that the difference was statistically significant.

## Results

### Sociodemographic characteristics of TB patients

A total of 250 questionnaires were distributed, and 250 valid questionnaires were returned. The valid questionnaire rate was 100.0% (250/250). The 250 TB patients were recruited from three sampled counties: Y County (*n* = 100, 40.0%), H County (*n* = 50, 20.0%), and T County (*n* = 100, 40.0%). Table 1 shows that most of the patients were male (69.2%), aged >60 years (45.2%), farmers (90.8%), had an education level of junior high school or below (85.2%), belonged to the Zhuang ethnic group (72.0%), were married (84.0%), had an annual household income of ≦20,000 RMB, were covered by the medical insurance for urban and rural residents (95.2%), were undergoing initial treatment (89.2%), and had negative etiological diagnosis (52.0%). Among the patients, 82.4% reported that they did not have any other chronic diseases, 72.0% did not experience diagnostic delay, 79.6% visited a doctor once before diagnosis, 55.2% had more than 7 days of hospitalization before diagnosis, 76.8% had 0 days of hospitalization after diagnosis, and 70.0% were not the highest income earners in their household.

**Table 1:** Sociodemographic characteristics of TB patients (*n* = 250).

Variables	Frequency	Percentage (%)
Gender		
Man	173	69.2
Woman	77	30.8
Age		
<=40	40	16.0
41 ~ 60	97	38.8
>60	113	45.2
Nationality		
Zhuang	136	54.4
Han and others	114	45.6
Education		
Junior high school or below	213	85.2
High school or above	37	14.8
Occupation		
Famer	227	90.8
Others	23	9.2
Marriage		
Unmarried	28	11.2
Married	210	84.0
Divorced or widowed	12	4.8
Annual household incomes (RMB)		
≦20,000	81	32.4
20,001∽40,000	47	18.8
40,001∽60,000	50	20.0
>60,000	72	28.8
Medical insurance		
For urban and rural residents	238	95.2
For urban employees		4.8
Treatment classification		
Initial treatment	223	89.2
Retreatment	27	10.8
Etiological diagnosis		
Negative	130	52.0
Positive	120	48.0
Other chronic diseases		
Yes	44	17.6
No	206	82.4
Delay in diagnosis		
Yes	70	28.0
No	180	72.0
Number of healthcare visits before diagnosis		
0	23	9.2
1	199	79.6
≧2	28	11.2
Hospitalization days before diagnosis		
0	75	30.0
1 ~ 7	37	14.8
>7	138	55.2
Hospitalization days after diagnosis		
0	192	76.8
1 ~ 7	30	12.0
>7	28	11.2
The highest earners		
Yes	75	30
No	175	70

### Costs of TB care

Table 2 shows that during the pre-diagnosis stage, post-diagnosis stage, and the entire course of TB care, the median costs per TB patient were 6048.2 RMB, 3467.9 RMB, and 12461.0 RMB, respectively; the median out-of-pocket payments per TB patient were 2501.5 RMB, 2323.8 RMB, and 6323.7 RMB, respectively. Direct medical costs constituted the largest component of total costs at each stage of TB care.

**Table 2:** Costs of TB care (RMB).

Variables	Minimum	Maximum	*M*	Q1	Q3
Before diagnosis					
Total costs	0.0	89076.0	6048.2	1234.0	10221.0
Direct medical costs	0.0	73000.0	5812.7	774.1	9133.6
Direct non-medical costs	0.0	6476.0	200.0	15.0	373.5
Indirect costs	0.0	20833.3	0.0	0.0	274.6
Out-of-pocket payments	0.0	459861.0	2501.5	876.9	5043.8
After diagnosis					
Total costs	815.2	292807.8	3467.9	2320.6	13799.1
Direct medical costs	519.2	55305.2	2825.6	2041.7	6083.4
Direct non-medical costs	0.0	4720.0	150.0	83.8	420.0
Indirect costs	0.0	283933.3	0.0	0.0	282.5
Out-of-pocket payments	5.0	289962.6	2323.8	1452.5	9683.9
The entire course of TB care					
Total costs	1808.1	331108.2	12461.0	7748.6	21990.2
Direct medical costs	1638.1	75305.2	9835.7	6580.5	14708.8
Direct non-medical costs	0.0	7148.0	405.0	241.8	772.0
Indirect costs	0.0	304766.7	0.0	0.0	2271.4
Out-of-pocket payments	594.1	478147.0	6323.7	3533.7	13630.4

### Analysis of factors influencing catastrophic costs due to TB

The incidence of catastrophic costs due to TB was 45.6% (114/250). Table 3 shows the results of the $x^{2}\;$ test. There were statistically significant differences in the incidence of catastrophic costs due to TB in patients with different education levels, annual household incomes, types of medical insurance, and lengths of hospitalization after diagnosis (*p* < 0.05). Patients with a junior high school education level or below, an annual household income of ≦20,000 RMB yuan, medical insurance for urban and rural residents, and hospitalization for more than 7 days after diagnosis had a higher incidence of catastrophic costs due to TB.

**Table 3:** Results of the univariate analysis of catastrophic costs due to TB.

Variables	Number of patients	Catastrophic costs		*X^2^*	*P*
		Frequency	Incidence (%)		
Gender				0.631	0.427
Man	173	76	43.9		
Woman	77	38	49.4		
Age				0.886	0.642
<=40	40	20	50		
41 ~ 60	97	46	47.4		
>60	113	48	42.5		
Nationality				0.067	0.796
Zhuang	136	61	44.9		
Han and others	114	53	46.5		
Education				4.409	0.036
Junior high school or below	213	103	48.4		
High school or above	37	11	29.7		
Occupation				2.348	0.125
Famer	227	107	47.1		
Others	23	7	30.4		
Marriage				1.869	0.393
Married	28	16	57.1		
Unmarried	210	92	43.8		
Divorced or widowed	12	6	50		
Annual household incomes (RMB)				61.676	0.000
≦20,000	81	63	77.8		
20,001 ~ 40,000	47	21	44.7		
40,001 ~ 60,000	50	19	38		
>60,000	72	11	15.3		
Medical insurance				4.254	0.039
For urban and rural residents	238	112	47.1		
For urban employees	12	2	16.7		
Treatment classification				0.016	0.898
Initial treatment	223	102	45.7		
Retreatment	27	12	44.4		
Etiological diagnosis				2.917	0.088
Negative	130	66	50.8		
Positive	120	48	40		
Other chronic diseases				0.474	0.491
Yes	44	18	40.9		
No	206	96	46.6		
Delay in diagnosis				0.759	0.384
Yes	70	35	50		
No	180	79	43.9		
Number of healthcare visits before diagnosis				0.608	0.738
0	23	9	39.1		
1	199	91	45.7		
≧2	28	14	50		
Hospitalization days before diagnosis				1.434	0.488
0	75	30	40		
1 ~ 7	37	17	45.9		
>7	138	67	48.6		
Hospitalization days after diagnosis				8.988	0.011
0	192	78	40.6		
1 ~ 7	30	17	56.7		
>7	28	19	67.9		
The highest earners				0.111	0.739
Yes	75	33	44		
No	175	81	46.3		

Multivariate analysis of catastrophic costs due to TB was performed using binary logistic regression. The dependent variable was whether catastrophic costs occurred (coded as 1 for yes, 0 for no). The independent variables included education level, annual household income, type of medical insurance, and number of hospitalization days after diagnosis, which were selected based on statistical significance (*P* < 0.05) in the univariate *χ^2^* tests. Table 4 shows the results of the binary logistic regression analysis. Annual household income and length of hospitalization after diagnosis were identified as significant factors influencing catastrophic costs due to TB (*p* < 0.05). Patients with an annual household income of ≦20,000 RMB and a hospitalization duration of more than 7 days after diagnosis had a higher risk of experiencing catastrophic costs due to TB. The ORs (95%CI) were 34.168 (12.742–91.625) and 7.488 (2.474–22.664), respectively. The regression model summary showed that the Nagelkerke *R^2^* value was 0.400, indicating that the independent variables included in the model had certain explanatory power for the outcome event (catastrophic costs), and the model fit was acceptable (at a moderate level).

**Table 4:** Results of the binary logistic regression analysis of catastrophic costs due to TB.

Variables	*B*	SE	Wald	*P*	OR (95%Cl)
Education					
Junior high school or below	0.21	0.46	0.20	0.652	1.231 (0.499 ~ 3.033)
High school or above	–				
Annual household incomes (RMB)					
≦20,000	3.53	0.50	49.23	0.000	34.168 (12.742 ~ 91.625)
20,001 ∽ 40,000	1.93	0.50	14.92	0.000	6.898 (2.589 ~ 18.383)
40,001 ∽ 60,000	1.67	0.50	11.18	0.001	5.287 (1.992 ~ 14.037)
>60,000	–				
Medical insurance					
For urban and rural residents	0.48	0.88	0.30	0.584	1.620 (0.288 ~ 9.114)
For urban employees	–				
Hospitalization days after diagnosis					
0	–				
1 ~ 7	1.55	0.49	10.00	0.002	4.725 (1.805 ~ 12.369)
>7	2.01	0.57	12.70	0.000	7.488 (2.474 ~ 22.664)
Constant	−3.154	0.977	10.419	0.001	0.043

Model fit statistics were as follows: number of observations (N) = 250, −2 Log likelihood = 255.706, Cox and Snell *R*^2^ = 0.299, and Nagelkerke *R*^2^ = 0.400.

## Discussion

In this study, three designated TB hospitals located in areas with different levels of per capita disposable income were selected. To the best of our knowledge, this is the first study to analyze the characteristics and influencing factors of TB costs from the perspective of patients and their families based on the total costs incurred due to TB. The findings provide critical evidence for achieving the WHO End TB Strategy target of zero TB-affected households facing catastrophic costs in southern China. The results showed that most TB patients in this survey were male, older adults, farmers, and initial treatment patients. These characteristics are closely aligned with those reported in the Guangxi provincial TB registry data (9, 13), suggesting that the study sample was representative and that the cost-related findings are generalizable to Guangxi’s high-burden TB population.

The results showed that the median total cost incurred over the entire course of TB per patient was 12461.0 RMB among the 250 TB patients. This was higher than the median total cost reported in the designated TB hospitals in Nanning, Guangxi (7,505 RMB), as reported by Zhao et al. (11). This indicated that the total cost incurred due to TB in Guangxi remained high, although it was lower than that reported in a related study conducted in four districts and counties of Chongqing (14, 156.8 RMB), as reported by Wu et al. (2). The results showed that the median out-of-pocket payment among TB patients was 6323.7 RMB, accounting for approximately half of the total cost incurred due to TB. This reflected the positive role of medical insurance reimbursement, civil assistance, and other subsidy measures in alleviating the financial burden of TB in Guangxi, but there is still room for further optimization (12). The median out-of-pocket payment per TB patients in in this study (6323.7 RMB) was higher than the figure reported in Guizhou Province (5,277 RMB) (14) but lower than the amounts reported in western China (8703.3 RMB) (15), eastern China (2389.5 USD) (16), and other similar surveys conducted in different regions (2, 4, 17). It was also lower than the figure reported in low- and middle-income countries worldwide (1,253 USD) (3). These differences may be related to factors such as the level of local economic development, medical insurance policies, and medical healthcare service capacity for TB diagnosis and treatment (18). These findings highlight that geographical and economic disparities in health equity across different regions and populations warrant further attention (4, 5).

The data showed that 90.8% of patients had their first visit at non-designated TB hospitals, some patients experienced a delay in diagnosis due to multiple visits to different hospitals, and the hospitalization rate before diagnosis was high (70%). In total, 55.2% of patients had more than 7 days of hospitalization before diagnosis. On the one hand, this might be attributed to the high reimbursement rates for inpatient care (typically 65–80% under China’s basic medical insurance schemes), which created a financial incentive and this facilitated agreement between non‑designated hospitals and patients on hospitalization as the initial intervention for symptomatic patients. This reimbursement disparity might lead to unnecessary hospitalization of TB-suspected cases, increased medical costs before diagnosis, and delayed referral to designated TB hospitals (19). On the other hand, examinations and medications before diagnosis were excluded from free TB service packages, and most treatment costs were not covered by medical insurance. These costs may also include expenses incurred from misdiagnosed treatments, thereby creating a significant financial barrier to early TB detection (20).

The substantial direct medical costs after diagnosis were primarily attributable to the treatment pathways in designated TB hospitals, including essential but non-reimbursed auxiliary services such as high-resolution CT scans and advanced biochemical profiling, as well as necessary adjunct medications, including hepatoprotective agents, immunomodulators, and second-line anti-TB drugs. These costs accounted for 76.8% of outpatient costs, but the proportion and amount of reimbursement provided by medical insurance for these costs were very limited, further aggravating the burden of medical costs. This finding is consistent with the conclusions of previous studies (2, 4, 21). It is suggested that, in TB prevention and control practice, efforts should continue to strengthen TB health education, improve awareness of designated TB hospitals, enhance supervision of TB examination and referral practices in non-designated TB hospitals, rationally control unnecessary hospitalization, examinations, and medication, and implement measures to reduce and compensate for direct medical costs due to TB (13, 15).

In this study, the incidence of catastrophic costs due to TB was 45.6% (114/250), which was lower than the reported figure in western China (52.82%) (14), as well as those reported in similar surveys conducted in other regions (2, 16, 18) and in low- and middle-income countries worldwide (54.9%) (3). However, it was higher than the rate reported in eastern China (27.4%) (16) and remains far from achieving the WHO End TB Strategy goal of zero catastrophic costs due to TB. This was related to the fact that TB in Guangxi was more common among rural residents, low-income populations, and older individuals, as well as the lack of public awareness regarding TB prevention and control (9). There is no doubt that the task of eliminating catastrophic costs due to TB remains challenging in Guangxi.

Annual household income and the length of hospitalization after diagnosis were identified as factors influencing catastrophic costs due to TB. Specifically, lower annual household income and longer hospitalization after diagnosis were associated with a higher risk of incurring catastrophic costs. Since most TB patients came from rural families with low income (90.8%), they faced substantial challenges in coping with the high economic burden of the disease. Previous studies have reported that such families often deplete important sources of income, borrow money, or sell assets to pay for TB treatment, leading to financial difficulties for the families and increasing the risk of catastrophic costs (14, 15, 22, 23).

Some studies also showed that delays in seeking healthcare were more common in patients with economic difficulties, and most of them might seek healthcare only at more severe stages of disease, leading to higher hospitalization rates and costs and subsequently increasing the risk of catastrophic costs (14, 15, 18, 23). Therefore, eliminating catastrophic costs due to TB requires a comprehensive strategy focusing on low-income and vulnerable households. This strategy should include establishing an early warning and monitoring system for high medical costs, developing a multi-tiered medical security system led by the government with multi-stakeholder participation, and creating sustainable mechanisms to prevent and address the poverty caused by TB, thereby promoting health equity through targeted measures (4, 23, 24).

At present, some counties and districts have initiated the “zero burden” pilot program for TB diagnosis and treatment, achieving initial success in alleviating the economic burden on patients with financial difficulties and exploring multi-party financing models in Zhejiang province, China (25).

In addition, there was a statistically significant difference in the incidence of catastrophic costs among patients with different education levels and different types of medical insurance. Patients with lower educational levels and those enrolled in medical insurance schemes for urban and rural residents had a higher incidence of catastrophic costs (15, 18–20, 26). Patients with lower educational levels might have fewer opportunities and channels to acquire TB knowledge and were more likely to experience delays in referral and treatment, resulting in a higher incidence of catastrophic costs (15, 18, 27).

Regarding insurance type, however, this association did not remain statistically significant after adjusting for other covariates. We attribute this attenuation primarily to confounding by household income. In China, enrollment in the two insurance schemes is largely determined by household registration status (hukou) and employment sector: urban employee medical insurance is typically available to formally employed individuals in urban areas with relatively higher and more stable incomes, whereas urban and rural resident medical insurance primarily covers rural populations, farmers, and urban informal sector workers who tend to have substantially lower household incomes. In our study, 90.8% of patients were farmers, and only 4.8% had urban employee insurance.

When annual household income was entered into the model alongside insurance type, the income variable—being the more proximal determinant of financial vulnerability—accounted for most of the variation in catastrophic cost risk, rendering the independent effect of insurance type non-significant. This pattern suggests that household income is not merely a confounder but rather lies on the causal pathway through which insurance type influences catastrophic costs, as lower-income households enrolled in resident insurance schemes face both higher absolute cost burdens and greater relative financial strain. Nonetheless, the small proportion of patients with urban employee insurance (4.8%) may also have contributed to the loss of statistical significance, and further research with larger and more balanced samples is needed to disentangle these effects (28).

Given that most TB patients could only participate in the medical insurance scheme for urban and rural residents and that current reimbursement levels differ substantially between the two schemes (19, 26), it remains necessary to explore and establish a differentiated medical insurance payment mechanism that conforms to the characteristics of financing, healthcare, and disease in both resident and employee insurance schemes (21, 29) and to continuously improve the capacity of medical insurance to protect urban and rural vulnerable groups from economic risks (15).

Furthermore, other influencing factors have been identified in related studies, such as gender, age, marital status, occupation, family size, and comorbidities (30); however, our study did not reach similar conclusions, which need to be verified in further research.

### Limitations and future directions

This study had several limitations. First, information regarding the course of medical care before diagnosis and the loss of income due to TB was self-reported by the respondents, which may have introduced recall bias. Second, the survey areas and the number of participants were limited, which could have potentially introduced some error in the results. Third, TB patients who did not receive treatment or discontinued treatment prematurely were not included in the study, which made it impossible to assess their economic burden. This exclusion might have led to an underestimation of the actual level of economic burden. Fourth, because drug-resistant TB patients were not included, the conclusions of this study should be generalized with caution. Further research with a larger sample size and a robust study design is recommended.

## Conclusion

Under the current “free diagnosis and treatment” policy and the basic medical insurance payment mechanism, TB patients still face high out-of-pocket costs and a high incidence of catastrophic costs, which might cause severe economic hardship and increase the risk of TB-related poverty for low-income groups. Costs incurred before diagnosis and direct medical costs accounted for a major proportion of total costs throughout the entire course of TB care, and the longer the hospitalization days after diagnosis, the higher the risk of catastrophic costs. As the current TB care policy remains insufficient in safeguarding patients from financial hardship, it is necessary to improve the TB economic risk protection mechanism for low-income vulnerable groups in rural areas, implement targeted financial and social support measures, strengthen supervision of standardized TB diagnosis and treatment practices, and establish a long-term mechanism for preventing and eliminating TB-related poverty.

## Data Availability

The original contributions presented in the study are included in the article/supplementary material, further inquiries can be directed to the corresponding author.
